# Standardization of rehabilitation program for post-apoplectic limb spasm treated by Tongjing Tiaoxing tuina and scalp acupuncture with physical therapy

**DOI:** 10.1097/MD.0000000000020368

**Published:** 2020-05-22

**Authors:** Qiongshuai Zhang, Yufeng Wang, Guangcheng Ji, Fang Cao, Guanyu Hu, Deyu Cong, Xiaohong Xu, Bailin Song

**Affiliations:** a Department of Acupuncture and Tuina, Changchun University of Chinese Medicine; b Department of Tuina, Traditional Chinese Medicine Hospital of Jilin Province; c Department of Rehabilitation, The Third Affiliated Hospital of Changchun University of Chinese Medicine, Changchun; d Department of Acupuncture, The 1st affiliated Hospital of Henan University of Chinese Medicine, Zhengzhou, China.

**Keywords:** limb spasm, modified Ashworth scales, rehabilitation, stroke, study protocol

## Abstract

**Background:**

Tong Jing Tiao Xing tuina (TJTX) is a Chinese massage method. Excising with scalp acupuncture (ESA) is a treatment combining scalp electroacupuncture with physical therapy (PT), and yinao fujian formula (YNFJ) is a Chinese oral herbal granule medicine. The combination of the 3 methods is called the “Zhishen Tiaoxing” (ZSTX) rehabilitation program, which is used as an alternative of limb spasm after stroke. There is little available evidence demonstrating its safety and efficacy.

**Methods:**

This will be a subject-blind, randomized controlled trial conducted in 3 medical centers. It will strictly follow the Standards for Reporting Interventions in Clinical Trials of Acupuncture, 2010. We will recruit 316 patients with limb spasm after stroke, 200 from the Affiliated Hospital of Changchun University of Chinese Medicine, Changchun, China, 80 from the Second Affiliated Hospital of Heilongjiang University of Chinese medicine, Harbin, China, and 36 from Huashan Hospital of Fudan University, Shanghai, China. A block randomization sequence stratified by centers will be generated using SAS Version 9.2 software (SAS Institute, Cary, NC, USA), which was performed at the Guangdong Provincial Hospital of Chinese Medicine's Key Unit of Methodology in Clinical Research. The treatment group is treated with TJTX (once a day), ESA (once a day), and oral YNFJ (twice a day). The control group will be treated with PT. Two groups of patients will be treated 5 sessions a week for 4 weeks, and there will be 6-month follow-up. The outcome evaluators will be blinded to patient grouping. The primary outcome will be modified Ashworth scales. The secondary outcome indexes will be the simplified Fugl–Meyer assessment scale, surface electromyogram root mean square value, modified Barthel index, stroke-specific quality of life scale, health scale of traditional Chinese medicine, visual analogue scale (VAS), and the Hamilton depression scale.

**Discussion:**

The Randomized Controlled Trial (RCT) mainly aim to evaluate the effectiveness and safety of traditional Chinese medicine rehabilitation program, by comparing the treatment of ZSTX with the PT for the treatment of limb spasm after stroke.

**Trial registration:**

Chinese Clinical Trial Registry: ChiCTR 1900024255. Registered on July 3, 2019.

## 1 Introduction

Stroke poses a serious threat to human health and life with high incidence and disability rates.^[1–4]^ It is the third most common cause of disability in the world^[5]^ and the number one killer in China.^[6]^ Spasticity, the incidence of which ranges from 17% to 43% in post-stroke patients^[7–9]^ is a common dysfunction, causing pain, affecting the free movement of limbs, and causing joint contractures.^[10–13]^ Spasticity after stroke is a major problem for rehabilitation. It is also a hot topic in international medical research. In 2016 AHA/ASA adult stroke rehabilitation treatment guidelines,^[14]^ oral drugs, normal limb position placement, and botulinum toxin injections were recommended. Nevertheless, there are some limitations in those treatments. Oral antispasmodic drugs act on the whole body but cannot selectively target the spasm site. The effect of botulinum toxin injection lasts no longer than 3 to 4 months, and it is necessary to determine whether continuous botulinum toxin injection is necessary.^[15]^ Although there is consensus among experts regarding intrathecal injection of baclofen, the sample size of the study was small and the technical requirements were high; therefore, it is not widely used.

Acupuncture, Chinese herbal medicine, and tuina have been used for a long time to treat post-stroke limb spasm in China. At present, a large number of hospitals in China use acupuncture, tuina, and other methods in addition to modern technologies for the rehabilitation of post-stroke limb spasm. Systematic reviews^[16–18]^ showed that Traditional Chinese Medicine (TCM) treatments were superior to other therapies; nevertheless, only a few provide sufficient clinical evidence, including high quality randomized controlled trial designs or large sample sizes. There are few high-quality and standardized studies of combinations of comprehensive TCM therapy rehabilitation methods.

Therefore, the purpose of this randomized controlled and assessor-blinded trial protocol is to determine the safety and efficacy of excising with scalp acupuncture (ESA) combined with Tongjing Tiaoxing tuina (TJTX) and Yinao Fujian formula (YNFJ) in rehabilitation of limb spasm after stroke.

## 2 Method and design

### 2.1 Study design

This study is a randomized, assessor-blinded, Sham-controlled, and parallel clinical trial. From August 2019 to December 2020, we will recruit 316 outpatients or inpatients suffering from limb spasm after stroke, who will be randomly divided into the treatment group (n = 158) and the control group (n = 158) using the central randomized system of clinical research of Guangdong Provincial Hospital of Chinese Medicine in Guangdong. Participants in the treatment group will receive ESA, TJTX, and YNFJ with fixed drug composition and fixed dose, while participants in the controlled group will receive PT.

**Figure 1:**
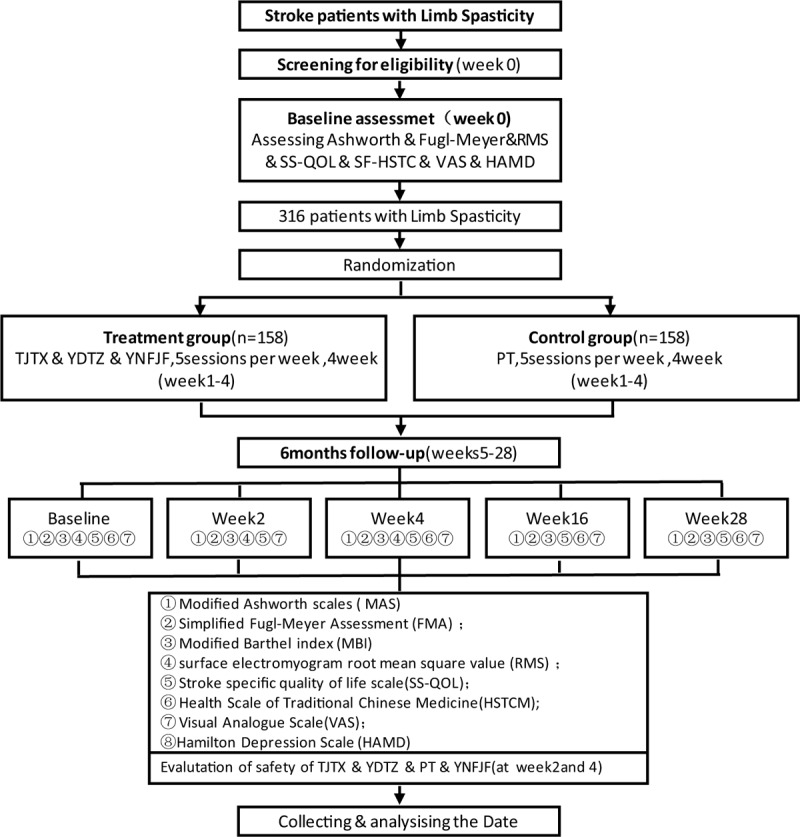
Trial flow chart.

During the study, we will the participants not to take other drugs that could affect the treatment of spasticity or other treatment methods that might affect the recovery of spasticity during the baseline, treatment, and follow-up periods. The basic drugs for stroke patients (i.e., antihypertensive drugs, hypoglycemic drugs, lipid-lowering drugs, and anti-platelet aggregation drugs) will be permitted. We will evaluate participants during the baseline period and at the second and fourth weeks after randomization (Fig. 1). This protocol will be reported according to recommendations for intervention trials SPIRIT 2013^[19]^ Statement: Defining Standard Protocol Items for Clinical Trials. The test process is designed according to the STICTA 2010^[20]^ report.

### 2.2 Participants

We will recruit 316 participants from the departments of Rehabilitation Medicine at 3 hospitals: Affiliated Hospital of Changchun University of Chinese Medicine (Changchun, China), The Second Affiliated Hospital of Heilongjiang University of Chinese Medicine (Harbin, China), and the Affiliated Huashan Hospital of Fudan University (Shanghai, China). Before treatment, we will draw complete blood counts, liver function tests, renal function test, and will obtain urinalyses, electrocardiograms, and other routine tests for the participants to select the patients to be included. In view of the limited mobility of patients with spasticity after stroke, we will recruit patients who are hospitalized at the 3 hospitals. For outpatients who want to participate in the study who meet the selection criteria, we will fully communicate with patients and their family members about everything regarding the trial face-to-face to ensure that they can participate in the entire study, including 6-month follow-up.

### 2.3 Recruitment

To recruit participants, we will publicize by such means of recruitment posters, rolling electronic screens, leaflets, radio programs, and WeChat public platforms. During recruitment, we will fully communicate with the patients, clearly informing them of the purpose, significance, test process, treatment methods, and possible benefits and risks of the study. We will then inform the patients and their guardians one-by-one of the contents of the informed consent form. When patients and their guardians completely agree with all the items in the consent form, they will sign it of their own free will.

### 2.4 Ethical issues

This study follows the Declaration of Helsinki. The trial has been approved by the relevant institutional review boards (IRBs). On June 13, 2019, the IRB of Affiliated Hospital of Changchun University of Chinese Medicine approved this study (cczyfyl2019 sz-022-1). On December 2, 2019, it passed the ethical review (irb-ap/sc-08/02.0) of the IRB of the Second Affiliated Hospital of Heilongjiang University of Chinese Medicine. Before the trial, we contacted the Chinese Clinical Trial Registry for registration and learned that participants can take part in the project only after they sign informed consent.

### 2.5 Inclusion criteria

Patients need to meet the following criteria: the diagnostic criteria of stroke^[21]^; symptoms of increased limb muscle tension, with the modified Ashworth Scale (MAS) score grade 1–2; the course of disease is within 2 weeks to 6 months; age 35–70 years, male or female; conscious and have stable vital signs; and participants or family guardians provide written informed consent.

### 2.6 Exclusion criteria

Participants will be excluded if they meet any of the following conditions: other diseases that may cause limb spasm; severe primary diseases of liver, kidney, hematopoietic system, endocrine system, or other complicating diseases that cannot be controlled well; audio-visual abnormalities, severe cognitive impairment, or mental diseases such that the patient cannot cooperate with examination and treatment; pregnancy or lactation; presence of a pacemaker; allergy to metal or severe needle syncope; blood diseases or coagulation disorders; and participation in other clinical studies at the same time.

### 2.7 Exit criteria

Participants can withdraw from the trial under any of the following circumstances: participants can withdraw at any time without any reason; death or other major adverse events or accidents occur due to disease development; participants cannot cooperate with the treatment during the treatment process, or change the treatment method in the study, or cannot provide data related to the study; participants who could not complete the 6 months follow-up for any reason.

### 2.8 Randomization and assignment hiding

Using the “central randomized system of clinical research” of Guangdong Provincial Hospital of Chinese Medicine, 316 subjects will be randomly assigned to the treatment group and the control group according to the ratio of 1:1 by the method of regional group randomization. The personnel of Central Randomization System of Clinical Research of Guangdong Provincial Hospital of Chinese Medicine will be responsible for the random distribution of the included patients but they will not participate in the clinical treatment operations. After the participants are grouped, the therapist will be informed which group the patients are divided into, and then he will treat the patients in the group.

### 2.9 Blinding method

Blinding methods will be applied to data statisticians and outcome evaluators throughout the trial. They will not able to obtain information regarding grouping and treatment of participants. Acupuncture and tuina belong to the external treatment of TCM, it is difficult to implement blind method for operators, so clinical therapists do not participate in data collection, statistics, and final outcome evaluation. For the treatment of patients, we will assign different patients to different treatment rooms where the beds are independent so as to avoid contact among patients. We separate personnel who separately in charge of random grouping, clinical treatment, data processing, and outcome evaluation completely, so as to ensure the authenticity of the outcome of the trial to the greatest extent possible.

### 2.10 Interventions

Acupuncture in the treatment group will be performed by acupuncture doctors who have received unified clinical training of scalp acupuncture therapy, have obtained Certificates of Acupuncture, and have had clinical experience for >5 years. The tuina operations are all performed by clinical tuina therapists who have received training in the TJTX technique and have clinical experience of >3 years. In the control group, PT will be performed by rehabilitation therapists who have received unified training of PT and have had rehabilitation working experience for >3 years. In the treatment group, scalp acupuncture and tuina are both widely used in the clinical rehabilitation of patients with spasticity in post-stroke patients in TCM hospital. However, in the Chinese stroke rehabilitation treatment guidelines (2011 edition),^[22]^ which is the latest guide, PT (which performed in the control group) is recommended for the treatment of limb spasm after stroke. Both groups will receive 20 sessions (5 sessions per week) of treatment in 4 weeks.

#### 2.10.1 Treatment group:

The treatment group will be treated with TJTX (30 minutes in total, 1/d) combined with MSA (6 hours in total, 1/d) and YNFJ (2/d).

##### 2.10.1.1 Excising scalp acupuncture therapy

Supplies: needles (0.35 mm in diameter, 40 mm in length Hwato Brand, Suzhou Medical Appliance Factory, China), alcohol swabs (6 cm × 6 cm, HAINUO Brand, Qingdao Hainuo Bioengineering Co., Ltd., China), electronic acupuncture treatment instrument (KWD-8081, The Great Wall Brand, Changzhou Wujin Great Wall Medical Equipment Co., Ltd., China), aseptic cotton bud (HAINUO Brand, Qingdao Hainuo Bioengineering Co., Ltd., China).

**Figure 2:**
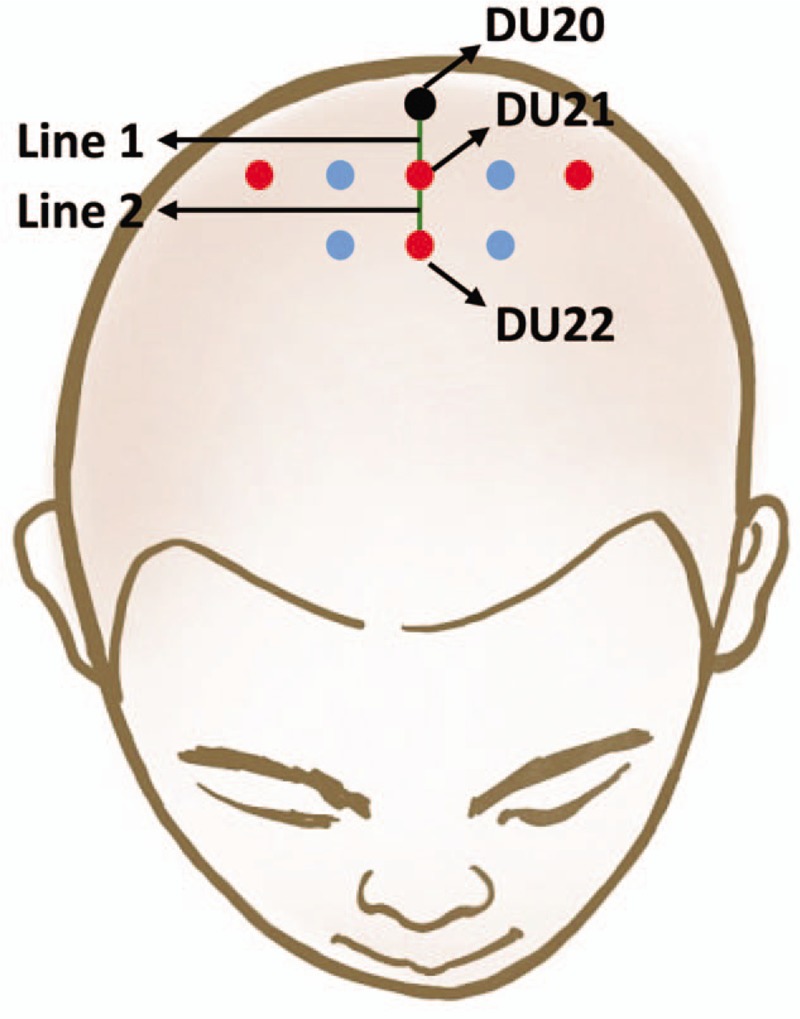
Acupoints of scalp acupuncture.

Acupoints: Positioning Qianding (DU21), Baihui (DU20), and XinHui (DU22) point (Fig. 2).

The patient lies supine, while the acupuncturist sterilizes Qianding (DU21) with alcohol swabs, inserts the needle into Qianding (DU21) at a 30° angle with the skin, then inserts the needle into the acupoint slowly and smoothly until it is under the cap aponeurosis, penetrating the depth of 25 to 40 mm with the direction to Baihui (DU20). The connection between Qianding (DU21) and Baihui (DU20) is named Line 1. From the left and right 1 cun and 2 cun points of Qianding (DU21), there will be needling along the direction parallel to line 1. From the Positioning XinHui (DU22) point, the acupuncturist inserts the needle into XinHui (DU22) with the same technique as above, penetrating towards Qianding (DU21). The connection between XinHui (DU22) and Qianding (DU21) is named Line 2. From the left and right 1 cun points of XinHui (DU22), the needle direction parallels line 2. Eight needles are used per session.

After the needles have penetrated, we will use electronic acupuncture treatment instrument to connect the acupoints on both sides of Qianding (DU21) and both sides of Baihui (DU20) point, with an intermittent wave of 80 Hz frequency and an intensity of 1 to 10 mA; 30 minutes later, we remove the electric acupuncture. We then maintain the needles in the head for 6 hours during which time the patients receive physical therapy, 5 sessions a week over 4 weeks.

##### 2.10.1.2 Tuina of Tong Jing Tiao Xing

**Figure 3:**
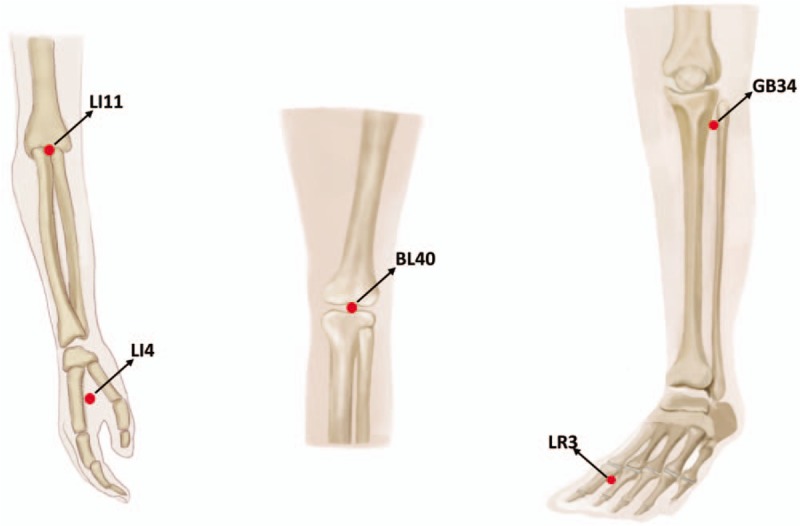
Acupoints of TJTX.

Acupoints: Hegu (LI4); Quchi (lI11); Weizhong (BL40); Yanglingquan (SP9); Taichong (LR3); Zhongwan (RN12) (Fig. 3).

Treatment process: the participant lies in the healthy-side-up position. The therapist kneads the spine with palm down to sacrum along DU channels 5 times (3 minutes); the participant then lies in the supine position. Therapist rolls on participant's upper and lower limbs along 3 yang meridians of hand and 3 yang meridians of foot of the affected side 3 times (the upper limbs for 3 minutes and the lower limbs for 5 minutes, total of 8 minutes). Then the therapist slowly flexes and stretches the affected shoulder, elbow, wrist, and metacarpophalangeal joints, then presses Quchi (lI11) and Hegu (LI4) points to promote elbow extension, wrist extension, and finger movement. The therapist slowly flexes and stretches the hip, knee, ankle, and metatarsophalangeal joints, pressing the Yanglingquan (SP9) point to promote ankle dorsiflexion, and pressing Taichong (LR3) (SP9) point to promote the extension of the metatarsophalangeal joint. The patient is turned lie in the healthy-side-up position, and the therapist presses the Weizhong (BL40) point to promote knee flexion, performing each of these movements 5 times (about 16 minutes); the therapist grasps upper and lower limbs top-down of the affected side 3 times (about 1 minute); and the therapist presses the Zhongwan (RN12) point with the palm, and gradually presses it with the breath until the operator feels the beat under the palm, and keeps the pressure constant for 30 seconds, and then releases the hand quickly when the participant breathes out. This is repeated 5 times (about 3 minutes).

##### 2.10.1.3 Yi Nao Fu Jian Fang

This prescription contains 9 Chinese herbal medicines, including Radix Paeoniae Rubra 5 g, Ligusticum chuanxiong 3 g, Puhuang 5 g, Pueraria 5 g, Sophora japonica 5 g, earthworm 5 g, safflower 3 g, Shencao 7.5 g, and Sanqi 4.5 g. Those are provided orally or nasally, 43 g per time, twice a day for 4 weeks.

##### 2.10.1.4 Physical therapy

After electroacupuncture, the participants will receive following treatments. Muscle spasm control: placement of good limb position; reflex inhibitory pattern (RIP), influence tension posture (TIP), and control key points in Bobath technology; using sensory stimulation of Rood technology, inhibition of spasm through corresponding sensory stimulation; promote the emergence of separation movement: further promote the separation movement of the affected side of the limb using nerve facilitation technology, sports relearning, and other training; therapeutic training; sitting balance training, standing balance training, walking training, up and down stairs training, and others.

#### 2.10.2 Control group

PT for the control group is the same as that of the treatment group.

### 2.11 Outcome measurements

Multiple dimensions are selected to evaluate the patients so as to determine the therapeutic effect of ESA combined with TJTX and YNFJ on spasticity post-stroke. Table 1 shows the times of evaluation of this study.

**Table 1:**
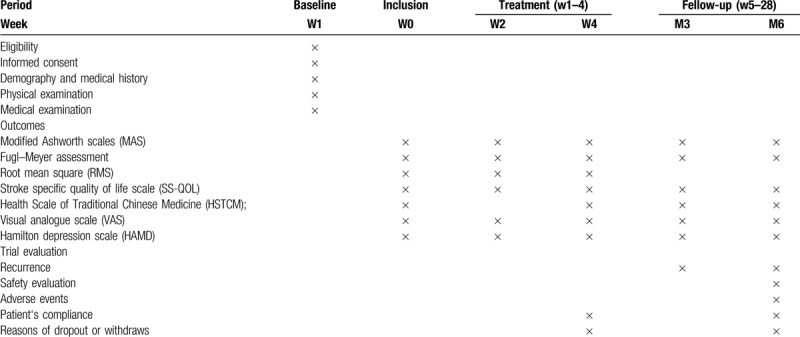
Research design schedule.

#### 2.11.1 Primary outcome

##### 2.11.1.1 The main outcome of this study is the modified Ashworth scale

The MAS will be used to evaluate muscle tone. The degree of spasm is divided into 0, I, I+, II, III, and IV levels according to the resistance felt by the evaluators during passive movement of the elbow and knee joints of hemiplegic patients in the resting state. The higher the score, the higher the degree of spasm.^[23,24]^ This will be evaluated at the—1st and at the 2nd and 4th weeks after randomization, and at the 3rd and 6th months after treatment.

#### 2.11.2 Secondary outcomes

##### 2.11.2.1 Simplified Fugl–Meyer assessment scale (FMA)

Functional rehabilitation will be assessed using the simplified FMA and the mini-mental state examination (MMSE). There are 50 items in the FMA, which are scored with 0 to 2 points, involving upper and lower limbs, with the highest score of 100 points. The higher the score, the better the motor function; the MMSE includes 5 items (orientation, memory, attention and calculation recall ability, and language ability) with a total score of 30. Higher score indicates better function. This is measured at the—1st and at 2nd and 4th weeks after randomization, and at the 3rd and 6th months after treatment.

##### 2.11.2.2 Surface electromyogram root mean square (RMS) value

Surface electromyogram (sEMG) is a neurophysiological measurement method in which electrode plates are attached to the skin to record the activity of skeletal muscle. As an effective objective quantitative evaluation index of spasticity state after stroke, the sEMG analysis system (Model: SA7550, Nanjing Weisi Medical Technology Co., Ltd.) is used in this study to evaluate the biceps and triceps of the affected side of upper humerus and RMS of sEMG of the quadriceps femoris and hamstring. This item is measured at the—1st and the 2nd and 4th week after randomization.

##### 2.11.2.3 Modified Barthel index (MBI)

The activity of daily living (ADL) is assessed by the MBI. There are 15 items in the Barthel Index with a total score of 0 to 100. A score of 0 indicates poor function, no independent ability, and all activities of daily living require help. A score of 100 indicates that the patient's basic activities of daily life are in good condition, and the help of others is not needed (the patient is not only able to control defecation, but can eat, dress, and bathe unaided). Lower scores demonstrate worse survival status. This will be evaluated at the—1st and at 2nd and 4th week after randomization, and at the 3rd and 6th months after treatment.

##### 2.11.2.4 Stroke specific quality of life scale (SS-QOL)

SS-QOL is used primarily to evaluate the life status of patients after stroke. This table includes 12 fields, including mood, energy, vision, language, social role, and others. There are 49 items in total. Each item is scored with a 5-level scoring method (1–5 points), and the scoring range is 0 to 245 points. Higher score indicates better living conditions. This is evaluated at the—1st and at the 2nd and 4th week after randomization, and at the 3rd and 6th month after treatment.

##### 2.11.2.5 Health scale of traditional Chinese medicine (SF-HSTC)

SF-HSTC consists of 28 items, which are classified into 5 aspects, including the social environment, physical function under natural environment, spirit, and ability of communication with people, adaptability of natural environment. The score range is 0 to 130. The higher the score, the worse the health status. This table requires the evaluation of the patients’ condition of the past month, so it is only evaluated during the baseline period and at the 4th week after randomization as well as the 3rd and 6th months after the end of treatment.

##### 2.11.2.6 Visual analogue scale (VAS)

VAS pain score is widely used for subjective pain evaluations. The modified evaluation involves marking the 2 ends of a 100-mm line as 0 and 10, respectively. Zero means no pain and 10 means the most pain. Patients can draw a mark on the line according to the subjective evaluation of their own pain. The distance from 0 to the marker indicates the degree of pain. This will be measured at the—1st and at the 2nd and 4th weeks after randomization, and at the 3rd and 6th months after treatment.

##### 2.11.2.7 Hamilton depression scale (HAMD)

The HAMD is the most generally used scale for clinical depression assessment. There are 17 items in the scale, and the score range is 0 to 52. The higher the score, the more severe the depression. This will be measured at the—1st and at the 2nd and 4th weeks after randomization, and at 3rd and 6th months after treatment.

### 2.12 Adverse events

During the experiment, we will ask the participants to report adverse events in the course of treatment, including dizziness, nausea, palpitation, collapse, pain, and itching at the acupuncture site, as well as bleeding and persistent pain at the acupuncture site or after acupuncture. The patient may have increased pain during the Tuina, or bruise, swelling, persistent pain of the limb after the Tuina. Nausea, vomiting, diarrhea, acid regurgitation, and other symptoms may occur after oral administration of traditional Chinese medicine. We also require the therapists to actively report stuck, bent, and broken needles in the acupuncture treatment and the skin abrasions of the patients during the tuina process. Researchers need to record the occurrence times, situations, grades, corresponding treatment methods, and the relationship between the time of adverse reactions and treatment measures in case report forms.

### 2.13 Sample size calculation

The sample size calculation will be conducted based on a study by White.^[25]^ This was a study using the modified Ashworth spasticity scale (MAS) as the main outcome, that showed that the total effective rate of physical therapy for spasticity of post-stroke was 45.5%.^[26]^ Our early clinical observation suggested that the total effective rate of the comprehensive rehabilitation treatment program of TCM in this study would be 65%. According to comparison of independent sample rates between 2 groups to calculate sample size: *α* = 0.05, *β* = 0.1, power = 90%, 132 participants in each group will be recruited. Considering an estimated 20% dropout rate, a total size of 158 cases in each group will be recruited.

### 2.14 Data collection and supervision

The project team has made SOPs for clinical research to ensure that the treatment is followed throughout the clinical research. At the same time, in accordance with the requirements of the standard for quality control and quality assurance of clinical research in TCM, a 4-level quality supervision system will be established. Each clinical research unit appoints 1 supervisor, each clinical center appoints one supervisor, and sets up a supervision committee to regularly monitor the data collected in clinical research, so as to ensure the authenticity and reliability of the project. A special data collector will record the patients’ personal data in the Case Report Form (CRF) forms. At least 5 completed CRFs from each clinical trial center shall be sent to the data manager in a timely fashion by the supervisor so as to establish a corresponding database. All data will be double entered using the data entry program run on a software platform. The data administrator shall ensure that the data in the CFR forms are completely and truly entered into the computer. The supervision team will supervise the entire process of information collection and input.

### 2.15 Data analysis contents and methods

Statistical analysis will be conducted by GPHCM's Key Unit of Methodology in Clinical Research. We will use bilateral test with a significance level of *α* = 0.05, performed using SPSS (SPSS, SPSS Inc., Chicago, IL) version 21.0.

For efficacy evaluation indexes, the data sets of per-protocol analysis (PP) and intention to treat analysis (ITT) will be used. A safety analysis set (SS) will be used for safety evaluation and analysis. Depending on the data type, appropriate statistical methods will be used. First, descriptive statistics will carried out for baseline characteristics of patients. The efficacy evaluation index data are repeated measurement data that can be used for the analysis of variance of repeated measurement. Due to the data missing values caused by withdrawal, the method of multiple filling will be used in ITT analysis. If necessary, the method of missing value processing provided by SPSS version 21.0 can be used to replace data, and various methods will be selected according to the actual situation. For safety evaluation analysis, we will test and compare the incidence of adverse reactions between 2 groups. The severity of adverse reactions and the degree of causality with treatment will be considered in comparison. If the number of adverse reactions is large, the relationship with treatment duration and baseline characteristics will be analyzed.

## 3 Discussion

Spasticity of limbs after stroke is a common complication after stroke. Spasticity occurs with high incidence and carries a high disability rate. There are several methods used in rehabilitation; nevertheless, their effects are not obvious. In China, acupuncture, tuina, oral Chinese herbal medicine, and other TCM methods are widely used for the treatment of spasticity after stroke. At present, there are relatively few clinical studies on acupuncture and tuina, and most of these studies involve acupuncture or tuina as single intervention methods. Most research content is not rigorous, the evaluation indexes are one-sided, and the sample sizes are small. However, in reality, the rehabilitation of patients often involves a combination of several rehabilitation methods; therefore, it is more realistic to study the effect of a rehabilitation program. We need to combine the advantages of various methods to develop a comprehensive and effective treatment plan to help patients recover limb function. For these reasons, in this study, we will integrate the currently effective methods in the field of TCM for the treatment of spasticity after stroke, and will attempt to standardize a rehabilitation program, and to evaluate its safety and efficacy using a rigorous experimental design.

The intervention measures adopted in this study have been verified in many hospitals for a long time, combining many years of clinical application and expert consensus. The Tuina of “TJTX” at the Rehabilitation Department of Affiliated Hospital of Changchun University of Chinese Medicine has shown good clinical effect for the treatment of limb spasm after stroke. “ESA” has been used for 12 years at the Department of Rehabilitation Medicine of the Second Affiliated Hospital of Heilongjiang University of Chinese medicine. “YNFJ” formula was invented by Professor Ren Jixue, a master of Chinese medicine, and it has been applied in the rehabilitation of patients after stroke. Nowadays, the drug is on the market.

In the present trial, because bleeding may occur related to acupuncture, patients with coagulation disorders will be excluded. Because most of the patients with spasticity after stroke have dyskinesia, to facilitate their daily treatment, we will recruit hospitalized patients. Outpatients who meet inclusion criteria must promise to be able to fully accept the trial study process.

The evaluation indexes of this study include 7 scales and surface electromyogram root mean square value (RMS). It is difficult to ensure that patients complete the scale with cooperation in the trial process. To encourage patients to complete each evaluation, researchers will list the name of patients who complete each evaluation, and promise that patients who complete each evaluation can obtain free treatment for 1 week after completion of the trial. Because the follow-up time of this study is 6 months, considering the limited activity of most patients, we will determine the follow-up method by communicating with patients before treatment: patients can complete the follow-up at the hospital, we will give each participant a transportation subsidy of 100 RMB each time; for participants who may not be able to complete the follow-up visit in the hospital, we will record patients’ home addresses, phone numbers (or those of their family members) in advance so as to perform the follow-ups as home visits. We encourage patients to come to the hospital as often as possible to complete follow-up.

There are some deficiencies in the design of this trial. Most of the interventions in this study are external treatments; therefore, it is difficult to implement blind treatment for the 2 groups of staff participating in the treatment. Therefore, we will arrange the 2 groups of participants in different treatment rooms, and each participant will be treated separately to avoid communication between the patients in the group.

This research is funded by the state key R&D program supported by the Ministry of science and technology of the Peoples’ Republic of China. The purpose of this study is to provide new and high-quality evidence for the treatment of limb spasm after stroke using a TCM rehabilitation program.

## Author contributions

**Conceptualization:** Deyu Cong, Yufeng Wang, Bailin Song.

**Funding question**: Deyu Cong.

**Investigation**: Qiongshuai Zhang, Guanyu Hu.

**Methodology**: Deyu Cong, Yufeng Wang, Guangcheng Ji, Qiongshuai Zhang.

**Project administration**: Deyu Cong, Yufeng Wang.

**Resources**: Qiongshuai Zhang, Guangcheng Ji.

**Writing** – **review and editing**: Bailin Song, Yufeng Wang.

**Writing** – **original draft**: Qiongshuai Zhang, Fang Cao, Xiaohong Xu, Guanyu Hu.
